# A comparative assessment of alternatives to the full-leg radiograph for determining knee joint alignment

**DOI:** 10.1186/1758-2555-4-40

**Published:** 2012-10-30

**Authors:** Amir M Navali, Leila Azhar Shekoufeh Bahari, Behrouz Nazari

**Affiliations:** 1 Department of Orthopaedic Surgery, Tabriz Medical Sciences University, Tabriz, Iran

**Keywords:** Knee, Alignment, Clinical, Radiographic, Measurement

## Abstract

**Background:**

The purpose of this study was to assess the concurrent validity of alternative measures of frontal plane knee alignment, namely the radiographic anatomic axis and two clinical measures in patients complaining of knee malalignment as compared with the mechanical axis on full-length radiograph of lower limbs.

**Methods:**

The knee-alignment angle was measured in 100 knees of 50 subjects with the chief complaint of frontal knee malalignment according to the following methods: lower-limb mechanical axis on radiograph, lower-limb anatomic axis on radiograph, distance between medial femoral condyles or medial malleoli using a calliper and lower-limb alignment using a goniometer. Data were analyzed using Pearson’s correlation coefficient and simple linear regression.

**Results:**

The anatomic axis best correlated with the mechanical axis (r = 0.93, P<0.001), followed closely by the intercondylar/intermalleolar distance measured by calliper (r = 0.89, P<0.001). Significant correlation was also found between the mechanical-axis angle and the lower limb axis measured by goniometer (r = 0.67, P<0.001).

**Conclusions:**

The anatomic axis on radiograph, the calliper method and to a lesser extent the goniometer measurement appear to be valid alternatives to the mechanical axis on full-leg radiograph for determining frontal plane knee alignment. These alternative measures have the potential to provide useful information regarding knee alignment and may increase the assessment of this parameter by clinicians and researchers.

## Background

Axial alignment of the lower extremities is critical with respect to determining which portion of articular cartilage is repeatedly exposed to body weight during gait and is an important consideration in many clinical situations, whether considering fracture reduction, total knee arthroplasty or deformity correction.

Frontal plane malalignment has important biomechanical consequences because it influences loading across the knee joint during weight bearing. In the neutrally aligned knee, the ground reaction force vector passes medially to the joint center, creating an adduction moment that increases medial compartment forces relative to lateral [1]. When the knee is malaligned in the varus direction, the moment arm for ground reaction force vector is increased, resulting in a higher adduction moment than that observed in the neutral knee. Valgus malalignment results in a more laterally positioned ground reaction force vector and increases forces across the lateral knee compartment. In knee osteoarthritis, malalignment of greater than 5 degrees in either a varus or valgus direction is associated with significantly greater functional decline over time when compared with less malaligned knees [2].

Despite the importance of identifying malalignment in patients with knee problems, assessment of malalignment remains problematic. The gold standard for assessment is the weight-bearing full-leg radiograph, which allows the mechanical axis of the lower limb to be determined. This radiograph exposes the patient to x-rays and is not feasible for many health care professionals and researchers who are unable to directly request radiographs funded by national health care systems. Furthermore, the evaluation of alignment from the radiograph requires extrapolation of the femoral and tibial mechanical axes using bony landmarks, thus it is somewhat time consuming for the clinician or researcher to determine [3].

Determination of knee alignment using the anatomic axis is possible from a single knee radiograph [4,5]. This is a cheaper alternative, exposes the patient to less radiation, and is a routine test ordered for many patients with knee malalignment [3]. Several clinical measures of knee alignment have been reported in the literature, including goniometry [6], visual observation [7], and calliper methods of measurement [8]. Although such methods offer great clinical application with regard to cost, simplicity of use, and speed of result, clinical measures of knee alignment need to be validated against the mechanical axis determined by radiography.

Few studies that have investigated the validity of clinical methods for measuring knee alignment have been conducted in patients with osteoarthritic knee malalignment [3,9-12]. The aim of the present study was to evaluate the concurrent validity of the radiographic anatomic axis and two clinical measures of frontal plane knee alignment both in patients with mild osteoarthritis and in young healthy individuals. Moreover, the correlation between these measures and the mechanical axis on radiograph, as a gold standard for determining knee alignment, was analyzed.

## Methods

In a prospective study, 73 patients with the chief complaint of knee malalignment (genu varum or genu valgum) who had a previously obtained long standing x-ray of the lower limbs were evaluated. Participants were excluded if they reported a previous hip or knee joint replacement, hip or lumbar spine arthritis or other pathology causing joint contracture, pelvic obliquity, knee joint subluxation, congenital anomalies, previous lower-limb surgery or a body mass index greater than 32 kg/m [2]. After exclusions, 50 patients (100 knees) with a mean age of 35 years (range: 14–60 years) were included in the analysis. The duration between obtaining the radiograph and performing the clinical examination was less than 4 months in all cases. All participants provided written informed consent prior to the study.

The mechanical axis was measured according to the method described by Sharma et al. [2] on a weight-bearing full length anteroposterior radiograph of the lower extremities, which were imaged from the pelvis to the ankle (a full-limb radiograph). The mechanical axis was defined as the angle of intersection of the femoral and tibial mechanical axes. To determine the mechanical axis of the femur, a line was drawn from the center of the femoral head to the center of the femoral intercondylar notch. Concentric circles, known as Mose circles [13] and imprinted on a transparent template, were superimposed on the femoral head to precisely locate its center. A second line from the center of the tibial spines to the center of the ankle established the mechanical axis of the tibia. Alignment was recorded to the nearest half degree. The gold standard criterion, the mechanical axis, was used as the reference standard, with angles <180° defined as varus alignment, and angles >180° defined as valgus alignment.

Using the same full-leg radiograph, the anatomic axis was determined based upon the methods of Moreland et al. [5]. The femoral anatomic axis was found by drawing a line from the center of the tibial spines to a point 10 cm above the tibial spines, midway between the medial and lateral femoral surfaces. For the tibial anatomic axis, a line was drawn from the center of the tibial spines to a point 10 cm below the tibial spines, midway between the medial and lateral tibial surfaces. The anatomic axis was defined as the angle formed by the intersection of these two lines. All radiographic measurements were performed by a single examiner (BN). Intraobserver reliability for measuring the mechanical and anatomic axis was calculated with use of a randomized test of ten pairs of imaging studies that were measured a few days apart. The correlation coefficient was graded as excellent (0.92).

Interobserver reliability for clinical measurement was assessed with use of Kappa agreement coefficient in ten pairs of clinical studies performed by two different observer and because of good correlation (0.90 for calliper method and 0.79 for goniometer method) a single investigator (AN) blinded to radiographic findings performed the clinical measurements of alignment in all patients. For all measures, participants stood with their weight distributed equally between the lower limbs, with knees extended to replicate positioning for mechanical alignment via radiograph. Participants adducted their lower limbs slowly until either the knees or ankles touched each other. When the medial malleoli touched first, the participant was classified as having varus malalignment. If the knees touched first, the participant was classified as having valgus malalignment. If knees and ankles touched simultaneously, alignment was recorded as neutral [6].

**Figure 1:**
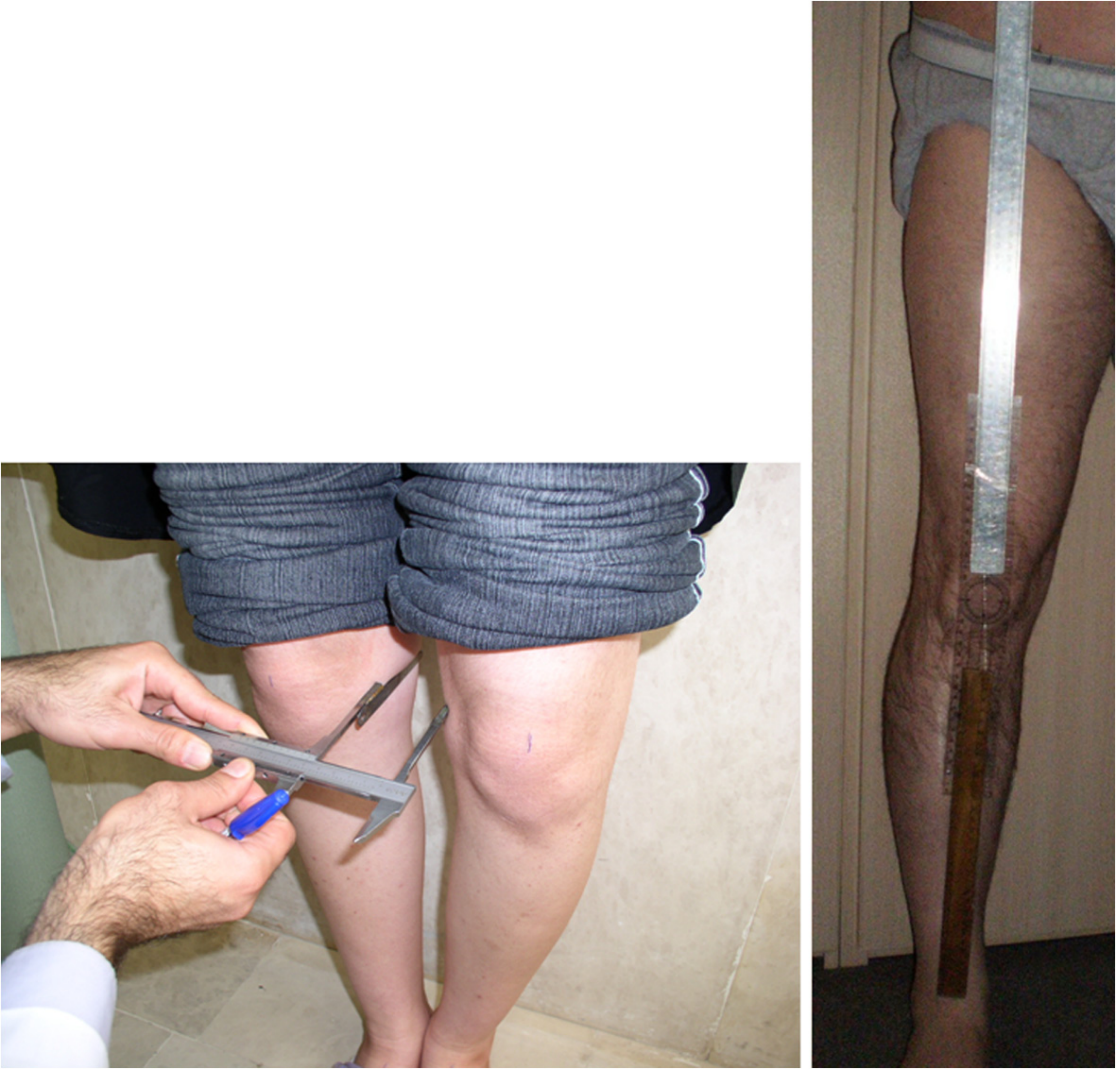
Determination of knee alignment using the calliper and goniometer methods.

In the first measurement the intercondylar and intermalleolar distances were measured with a caliper marked in 0.5-mm increments based on the methods of Cibere et al. [6]. In participants with varus knees the distance between the medial femoral condyles (Figure 1) and for valgus knees the distance between the medial malleoli was recorded. Varus and valgus malalignment were scored as negative and positive respectively.

In the second measurement the alignment of the lower limbs was recorded using a long-arm goniometer as described by Cibere et al. [6]. Participants stood on the foot maps. The axis of the goniometer was positioned over the center of the patella, and the arms were aligned with the anterior superior iliac spine above the knee and with the center of the ankle joint below the knee (Figure 1). Angles were recorded to the nearest degree, with 0° regarded as neutral.

### Statistical analysis

Data were analyzed using the SPSS version 13. Normal distribution of data was checked using Kolmogorov-Smirnov test and Q-Q plot. Correlations between mechanical alignment and the tested alignment methods were determined using Pearson’s correlation coefficients. Simple linear regression was used to develop regression equations for statistically significant relationships. Correlation coefficients of 0.5–0.75 were regarded as good, and values >0.75 were regarded as excellent [14]. P values less than 0.05 were regarded as statistically significant.

## Results

Patient characteristics are presented in Table 1. Thirty one (62%) participants had a varus mechanical axis, 15 had valgus (30%), and four had neutral (8%) alignment. The entire cohort demonstrated a mean varus malalignment of 4.1° from the neutral position, based on the mechanical axis on radiograph. Alignment characteristics of the cohort according to each measurement technique utilized are presented in Table 2.

**Table 1:** Patients characteristics*

**Characteristic**	**Value**
**Sex, male/female**	19/31
**Age (year)**	34.7 ± 15.2
**Height (meter)**	1.63 ± 0.15
**Weight (kg)**	66.7 ± 16.4
**Body Mass Index (kg/m2)**	23.26 ± 2.9

* Values are the mean ± SD unless otherwise indicated.

**Table 2:** Alignment characteristics according to each measurement procedure

**Method mean ± SD**	**95% Confidence interval**
**Mechanical axis, °† 175.9 ± 11.3**	173.73 - 178.19
**Anatomic axis, °† 179.1 ± 12.9**	176.53 - 181.65
**Calliper method, mm† −11.0 ± 81.3**	-27.13 – 5.11
**Goniometer, °† 178.1 ± 9.4**	176.21 – 179.93

^†^ Lower values represent a more varus knee alignment.

**Figure 2:**
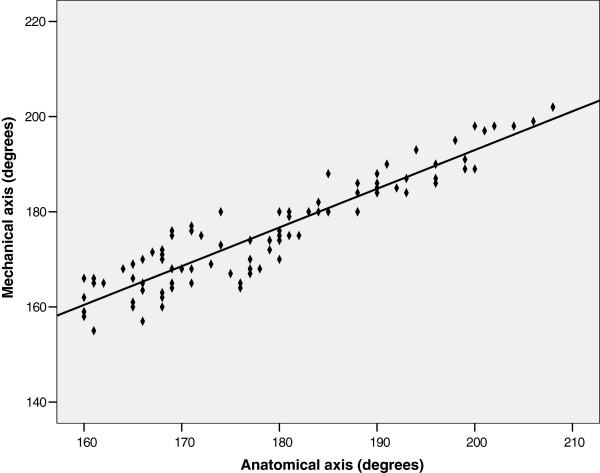
Scatterplot depicting the relationship between the anatomic axis and mechanical axis (n = 100).

There was a good correlation between the anatomic axis and the mechanical axis (r = 0.932, *P* <0.0001) (Figure 2). Regression analysis defined this relationship according to the following equation: mechanical axis = 0.814 (anatomic axis) + 30.244.

**Figure 3:**
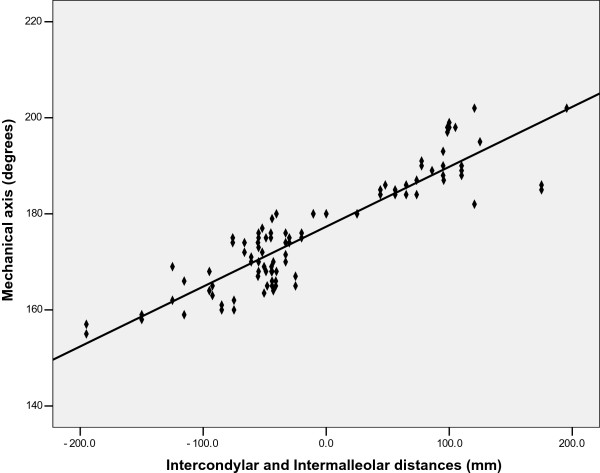
Scatterplot depicting the relationship between the Intercondylar and Intermalleolar distances (Calliper method) and the mechanical axis (n =100).

A good correlation with the mechanical axis was also observed for the calliper method (r = 0.899, *P* < 0.0001) (Figure 3). The relationship between the calliper reading and the mechanical axis was defined with the following equation: mechanical axis = 0.125 (calliper reading) + 177.333.

**Figure 4:**
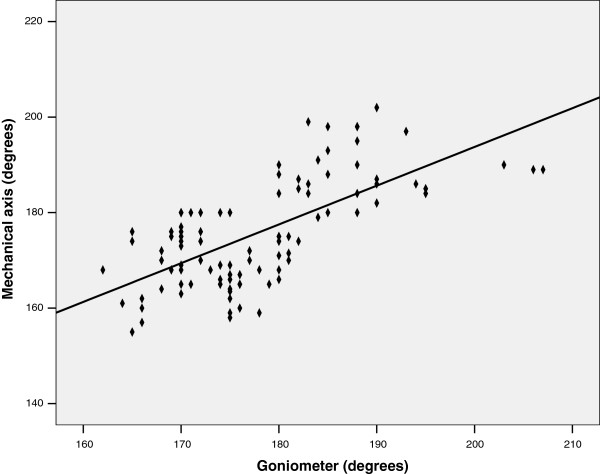
Scatterplot depicting the relationship between the goniometer method and the mechanical axis (n =100).

The goniometer method was found to have a weaker, although still statistically significant, relationship with the mechanical axis (r = 0.674, *P* < 0.0001) (Figure 4), generating a relationship defined with the following equation: mechanical axis = 0.811 (goniometer angle) + 31.461.

## Discussion

In the present study, we set out to validate more clinically accessible measures of knee alignment against the gold standard criterion of the mechanical axis measured on a full-limb radiograph. This study evaluated the criterion-related validity of three alternative measures for assessing malalignment, as compared with the mechanical axis. Anatomic axis measured on a standing radiograph was evaluated as an alternative radiographic measure of coronal alignment and best correlated with the mechanical axis (r = 0.93). According to numerous studies this measurement is best performed in a standing radiograph [3,10,11,15]. Importantly, intercondylar and intermalleolar distances (calliper method) were also shown to be valid indicators of the mechanical axis (r = 0.89). Although the goniometer method significantly correlated with the mechanical axis, this relationship was weaker than that demonstrated by the calliper method (r = 0.67).

Our study had several limitations. There might be a potential for selection bias as we did not include individuals without complaints of lower limb deformity in our study. Another limitation of our study is that the mechanical and anatomical axes were measured on the same radiograph. This means that there is no repositioning between the two measurements which may skews our results towards to a better correlation. To prevent further exposure to X-ray we did not order an additional short radiograph of the knee joints.

Concerning the limitation of our measurement techniques it should be noted that excessive soft tissue at the medial knee in obese patients may lead to a false classification of valgus malalignment in the caliper method, even though the underlying skeletal structure are actually in varus. Therefore, we excluded very obese patients in our study (BMI > 32 kg/m [2]). With regard to the use of a long-arm goniometer, the bony landmarks are sometimes difficult to locate especially in obese patients and positioning of the goniometer may be inaccurate. Consequently the results of clinical measurements should be interpreted with caution in obese individuals.

Few studies have investigated the validity of clinical methods for measuring knee alignment in patients with knee malalignment (Table 3). In their study comparing the validity of alternative measures of frontal plane knee alignment in individuals with medial knee osteoarthritis, Hinman et al. reported that the anatomic axis best correlated with the mechanical axis (r = 0.88, *P* < 0.0001), followed closely by the inclinometer method (r = 0.80) [3]. Other clinical measures of alignment that were significantly associated with the mechanical axis were the calliper method, the plumb-line method, and visual observation (r = 0.76, 0.71 and 0.52, respectively). They could not be able to show a correlation between the goniometer method and the mechanical axis. Our findings for anatomic axis and calliper methods were similar to these results but we could obtain a correlation between the goniometer measurement and mechanical axis which may be due to a better positioning with the use of a goniometer with very long arms that could sit exactly over the bony landmarks (Figure 1). In a similar study of osteoarthritic patients, Kraus et al. found significant correlations between the mechanical-axis angle and the anatomic-axis angle measured by each of the three methods: goniometer (r = 0.70, *P* < 0.0001), anatomic PA axis (r = 0.75), and anatomic AP axis (r = 0.65) [9].

**Table 3:** Summary of correlation coefficients (r) of alternative measures of frontal plane knee alignment relative to radiographic mechanical axis in recent major studies (p < 0.001)

**Study**	**Anatomic axis**	**Calliper**	**Goniometer**	**Inclinometer**	3D*
**Hinman et al.**[3]	0.88	0.76	No correlation	0.80	-
**Kraus et al.**[9]	0.75	-	0.70	-	-
**Mc Daniel et al.**[10]	0.74	-	0.74	-	-
**Sheehy et al.**[15]	0.88-1.00	-	-	-	-
**Vanwanseele etal.**[12]	-	-	-	0.83	0.93

*Three-dimentional Gait Analysis.

Mc Daniel et al. also compared the anatomic axis and a goniometer method with the mechanical axis in a cohort with symptomatic knee osteoarthritis [10]. In their study, anatomic axis was measured using a semiflexed knee radiograph. Similar to our findings, the authors demonstrated a significant correlation between the anatomic and mechanical axes (r = 0.74). They also reported a strong correlation between their goniometer method and the mechanical axis (r = 0.72). The measurement of knee-alignment angle by goniometer has been shown to be highly reproducible when performed by rheumatologists in the evaluation of patients with osteoarthritis [6].

Predictive validity of the anatomic axis on an extended-knee radiograph has previously been demonstrated by Cicuttini et al. who demonstrated that baseline anatomic axis was correlated with change in cartilage volume over time [11].

In a recent study Sheehy et al. showed that the correlations between hip-knee-ankle angle and femoral shaft-tibial shaft angle were excellent (range 1.00-0.88) but less correlation was noticed using progressively shorter shaft lengths [15].

The calliper method (intercondylar and intermalleolar distances) was also observed to be a valid measure of the mechanical axis. This method has been reported with respect to knee osteoarthritis in terms of its reliability and validity [3,6].

In another study Vanwanseele et al. assessed the validity of the hip-knee-ankle angle measured statically during three-dimensional (3-D) gait analysis and the tibial angle using an inclinometer compared with the mechanical axis on radiographs and suggest the inclinometer and 3-D gait analysis are valid ways to estimate mechanical alignment of the knee [12].

Advantages of the knee radiograph over the full-leg radiograph, which is the gold standard method, include reduced cost to the patient, researcher, and/or health care system; reduced exposure of the patient to ionizing radiation (particularly around the pelvis); and no problem with lack of a long graduated-grid cassette hampering determination of bony landmarks [3]. The knee radiograph forms part of the standard radiographic examination for knee problems, therefore extra radiographs just to determine the knee alignment, are not needed. In contrast to the radiographic methods, the goniometer and calliper methods of measuring alignment generate instant results, are quick to administer, are inexpensive (involving no cost to the patient/health care system and only a small outlay by the clinician/researcher to purchase the equipment required initially), and do not expose the patient to radiation [3].

Kawakami et al. showed that limb rotation affects the measurement of limb alignment and creates more measurement variability of the anatomic axis than of the mechanical axis [16]. As a result, inaccurate rotational positioning during clinical and radiographic evaluations may be misleading. On the other hand, due to weaker correlation observed with the mechanical axis, there is a greater risk of misclassification when using these clinical measures of alignment rather than the anatomic axis.

The very limited numbers of studies in the literatures which have addressed the validity of clinical methods for measuring knee alignment mandates the conduct of further studies to determine whether significant misclassifications using clinical methods of measurement do in fact occur, and to determine their consequences. Clinical measure of alignment is probably not a substitute for radiographic measures in surgical procedures. Therefore, we recommend that full-length radiographs be used whenever an accurate estimation of hip-knee-ankle angle is required.

In summary, we found that alternative methods of measuring knee alignment, which entail little or no radiation exposure, surprisingly correlated well with the knee-alignment angle measured on the more cumbersome and costly full-limb radiograph that includes pelvic irradiation. This study demonstrated that the radiologic anatomic axis and measurement of intercondylar and intermalleolar distances are relatively valid measures of determining knee alignment. Clinically, such methods may enhance the assessment of this important parameter by clinicians and researchers. Future research should evaluate the predictive validity of the clinical measures with regard to early disease detection and progression.

## Competing interests

The authors declare that they have no competing interests.

## Authors’ contributions

AMN conceived and designed the study, performed the measurements and assisted with writing. LAS analyzed the data, drafted the manuscript and assisted in revising the manuscript. BN assisted with conception of the study, designed the measurement devices and assisted in performing measurements and revising the manuscript. All authors read and approved the final manuscript.

## References

[B1] Schipplein OD Andriacchi TP Interaction between active and passive knee stabilizers during level walking J Orthop Res 1991 9 113 119 10.1002/jor.1100090114 1984041

[B2] Sharma L Song J Felson DT Cahue S Shamiyeh E Dunlop DD The role of knee alignment in disease progression and functional decline in knee osteoarthritis [published erratum appears in JAMA 2001; 286:792] JAMA 2001 286 188 195 10.1001/jama.286.2.188 11448282

[B3] Hinman RS May RL Crossley KM Is There an Alternative to the Full-Leg Radiograph for Determining Knee Joint Alignment in Osteoarthritis? Arthritis Rheum 2006 55 2 306 313 10.1002/art.21836 16583430

[B4] Hsu RW Himeno S Coventry MB Chao EY Normal axial alignment of the lower extremity and load-bearing distribution at the knee Clin Orthop Relat Res 1990 255 215 227 2347155

[B5] Moreland JR Bassett LW Hanker GJ Radiographic analysis of the axial alignment of the lower extremity J Bone Joint Surg Am 1987 69-A 745 749 3597474

[B6] Cibere J Bellamy N Thorne A Esdaile JM McGorm KJ Chalmers A Reliability of the knee examination in osteoarthritis: effect of standardization Arthritis Rheum 2004 50 458 468 10.1002/art.20025 14872488

[B7] Magee D Orthopedic physical assessment 1992 2 Philadelphia: W.B. Saunders

[B8] Jonson SR Gross MT Intraexaminer reliability, interexaminer reliability, and mean values for nine lower extremity skeletal measures in healthy naval midshipmen J Orthop Sports Phys Ther 1997 25 253 263 9083944 10.2519/jospt.1997.25.4.253

[B9] Kraus VB Vail TP Worrell T McDaniel G A Comparative Assessment of Alignment Angle of the Knee by Radiographic and Physical Examination Methods Arthritis Rheum 2005 52 6 1730 1735 10.1002/art.21100 15934069

[B10] McDaniel GE Vail TP Kraus VB Comparison of knee alignment angle by x-ray and goniometer [abstract] Arthritis Rheum 2004 50 Suppl 9 S343

[B11] Cicuttini F Wluka A Hankin J Wang Y Longitudinal study of the relationship between knee angle and tibiofemoral cartilage volume in subjects with knee osteoarthritis Rheumatology (Oxford) 2004 43 321 324 14963201 10.1093/rheumatology/keh017

[B12] Vanwanseele B Parker D Coolican M Frontal Knee Alignment Three-dimensional Marker Positions and Clinical Assessment Clin Orthop Relat Res 2009 467 504 509 10.1007/s11999-008-0545-4 18841432 PMC2628531

[B13] Mose K Methods of measuring in Legg-Clave-Perthes disease with special regard to the prognosis Clin Orthop 1980 150 103 109 7428206

[B14] Portney L Watkins M Foundations of clinical research: applications to practice 2000 2 Upper Saddle River (NJ): Prentice Hall Health

[B15] Sheehy L Felson D Zhang Y Niu J Lam YM Segal N Lynch J Cooke TD Does measurement of the anatomic axis consistently predict hip-knee-ankle angle (HKA) for knee alignment studies in osteoarthritis? Analysis of long limb radiographs from the multicenter osteoarthritis (MOST) study Osteoarthritis Cartilage 2011 Jan 19 1 58 64 10.1016/j.joca.2010.09.011 20950695 PMC3038654

[B16] Kawakami H Sugano N Yonenobu K Yoshikawa H Ochi T Hattori A Effects of rotation on measurement of lower limb alignment for knee osteotomy J Orthop Res 2004 22 1248 1253 10.1016/j.orthres.2004.03.016 15475205

